# Explaining age disparities in tuberculosis burden in Taiwan: a modelling study

**DOI:** 10.1186/s12879-020-4914-2

**Published:** 2020-03-04

**Authors:** Han Fu, Hsien-Ho Lin, Timothy B. Hallett, Nimalan Arinaminpathy

**Affiliations:** 1 MRC Centre for Global Infectious Disease Analysis, Department of Infectious Disease Epidemiology, School of Public Health, Imperial College London, London, W2 1PG UK; 2 Institute of Epidemiology and Preventive Medicine, College of Public Health, National Taiwan University, Taipei, Taiwan

**Keywords:** Tuberculosis, Age, Elderly, Modelling, Epidemiology, Taiwan

## Abstract

**Background:**

Tuberculosis (TB) burden shows wide disparities across ages in Taiwan. In 2016, the age-specific notification rate in those older than 65 years old was about 100 times as much as in those younger than 15 years old (185.0 vs 1.6 per 100,000 population). Similar patterns are observed in other intermediate TB burden settings. However, driving mechanisms for such age disparities are not clear and may have importance for TB control efforts.

**Methods:**

We hypothesised three mechanisms for the age disparity in TB burden: (i) older age groups bear a higher risk of TB progression due to immune senescence, (ii) elderly cases acquired TB infection during a past period of high transmission, which has since rapidly declined and thus contributes to little recent infections, and (iii) assortative mixing by age allows elders to maintain a higher risk of TB infection, while limiting spillover transmission to younger age groups. We developed a series of dynamic compartmental models to incorporate these mechanisms, individually and in combination. The models were calibrated to the TB notification rates in Taiwan over 1997–2016 and evaluated by goodness-of-fit to the age disparities and the temporal trend in the TB burden, as well as the deviance information criterion (DIC). According to the model performance, we compared contributions of the hypothesised mechanisms.

**Results:**

The ‘full’ model including all the three hypothesised mechanisms best captured the age disparities and temporal trend of the TB notification rates. However, dropping individual mechanisms from the full model in turn, we found that excluding the mechanism of assortative mixing yielded the least change in goodness-of-fit. In terms of their influence on the TB dynamics, the major contribution of the ‘immune senescence’ and ‘assortative mixing’ mechanisms was to create disparate burden among age groups, while the ‘declining transmission’ mechanism served to capture the temporal trend of notification rates.

**Conclusions:**

In settings such as Taiwan, the current TB burden in the elderly may be impacted more by prevention of active disease following latent infection, than by case-finding for blocking transmission. Further studies on these mechanisms are needed to disentangle their impacts on the TB epidemic and develop corresponding control strategies.

## Introduction

Global tuberculosis (TB) incidence and mortality have been declining in recent decades, with continuous efforts in improving case detection and treatment outcomes [1]. However, in many countries with intermediate TB burden, where the annual incidence lies between 10 and 100 per 100,000 population, a wide age disparity in TB burden is observed (Additional file 1: Fig. S1.1). For example, in South Korea, a substantially high TB incidence is reported among those older than 65 years old [2]. A similar age disparity is also reported in Hong Kong, where the age-specific TB notification rate is as large as 100 times among people older than 85 years old, compared to children less than 15 years old [3]. As life expectancy increases along with population ageing, these age disparities in TB burden may place an increasing need for prioritising TB control measures among the elderly, especially when countries are approaching the goal for TB elimination.

Age is a crucial factor in shaping TB epidemiology. Symptoms, disease progression risks, and treatment outcomes of TB patients are known to vary with age, probably caused by changes in the immune response with age [4–7]. Age-assortative social mixing patterns may contribute to different TB transmission risks across ages [8], as seen in other respiratory diseases [9, 10]. Overall, a systematic understanding of the driving mechanisms behind the large age disparities in TB burden would be valuable for control planning. For example, if concentrated TB burden in older age groups is due to a high risk of disease progression from remote infection, this suggests that measures to prevent reactivation, such as treating latent TB infection in the elderly, could be important. If, however, these disparities could be explained by preferential mixing between the elderly, measures to early diagnose and treat TB could be more impactful.

In the present study, we focus on the example of Taiwan, where has seen rapid growth in living standards and nationwide coverage of high-quality, publicly funded healthcare services [11]. In this setting, the age-specific TB notification rates in 2016 were 1.6 per 100,000 population amongst those under the age of 15, contrasting with 185.0 amongst those over the age of 65 [12]. We proposed and compared three mechanism hypotheses to explain these age disparities: (i) *Immune senescence* - due to a weakening immune system with older age, those with latent TB infection have an increased risk of developing TB as they age. (ii) *Declining transmission -* the force of TB transmission, despite being high in earlier generations, has undergone a rapid decline in recent decades owing to the improvement of living conditions and health services. Consequently, older generations are those most likely to have been exposed to TB, and this risk decreases sharply with declining age. (iii) *Age-specific assortativity* - elders have more contact with one another than with adults and children; any incident TB in elders therefore tends to cause infections in the same age group, with limited ‘spillover’ of infections to younger ages. We note that by itself, this mechanism does not necessarily drive a high burden in the elderly. Its potential value is to complement the other two mechanisms, to accentuate any disparity they produce.

We incorporated these mechanisms into a mathematical model of TB transmission dynamics. We evaluated the performance of these different mechanisms, acting alone and in combination, in capturing key features of TB epidemiology in Taiwan.

## Methods

### Model description

We developed a compartmental transmission model, where individuals are divided into three age groups: children (< 15 years old), adults (15–64), and elders (≥65). To replicate the historical demographics in Taiwan, we first executed the model in the absence of TB transmission dynamics with per-capita birth rates and age-specific death rates between 1900 and 2016 [13]. We employed the least-squares method to capture the aggregated population sizes and proportions of children, adults, and elders over time [13], by modifying the initial population size and transition rates between the three successive age groups. The best-fitting demographic parameters were then carried over to model TB transmission.

**Fig. 1:**
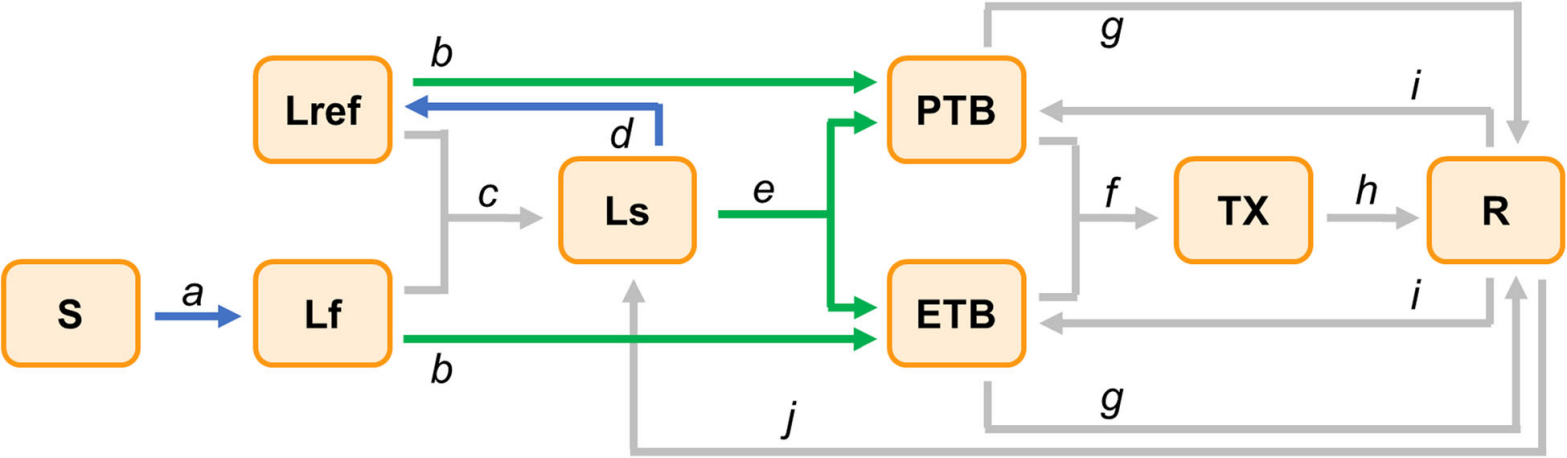
Schematic diagram of TB dynamic model. This is a simplified model structure of TB natural history within a single age group. Each box represents mutually exclusive disease states (S-susceptible, Lf-latent with fast progression, Lref-latent with fast progression from reinfection, Ls-latent with slow progression, PTB-pulmonary TB disease; ETB-extrapulmonary TB disease, TX-under treatment, and R-recovered). Arrows denote transitions between two states, with blue and green ones respectively highlighting the ‘infection’ and ‘progression’ processes that the proposed mechanisms modify (a-infection, b-primary progression, c-stabilisation from Lf/Lref to Ls, d-reinfection, e-reactivation, f-treatment initiation, g-natural recovery, h-treatment completion, i-relapse, j-stabilisation from R to Ls). For simplicity, birth, natural death and death during treatment are not shown in the figure

In the transmission model, within each age group, eight mutually exclusive compartments were constructed to represent the natural history of TB (Fig. 1). The state of latent TB infection is described by ‘fast-latent’, ‘fast-latent following re-infection’, and ‘slow-latent’ compartments, according to different risks of developing TB disease [5, 6, 14]. We also distinguished pulmonary and extrapulmonary TB in the model, with only pulmonary TB being infectious. TB cases may leave their active state by treatment initiation, self-recovery, or by disease-specific mortality rate, excess to the background mortality rate. We assumed treatment outcomes to be either cure or death, with failure accounting for < 2% in all age groups [15]. Post-treatment recurrence of TB was assumed to occur in the first 3 years after being cured, but after that, recovered individuals show the same hazard rate of developing the disease as those in the ‘slow-latent’ state [16].

### Base model (m0)

We refer to the simplest model, in which none of the proposed mechanisms is included, as the base model. The base model incorporates some age-specific parameters, including TB treatment outcomes [15], proportions of extrapulmonary TB among all cases [17], and recurrence rates [16], but otherwise treats all age groups equally (Additional file 1: Table S2.2). These values were assumed constant over time in the base model, while the force of TB infection varies in time, as:

$$ {\lambda}_{m0}(t)={\beta}_{m0}\ \frac{PTB(t)}{N(t)}, $$

which is proportional to pulmonary TB prevalence *PTB*(*t*), relative to a population *N* at a given time *t*. The infection rate *β*_*m*0_ was assumed to be independent of age and constant over time in the base model. In addition, the primary progression rate and reactivation rate in the base model were also assumed to be age-independent. For all age groups, we applied estimates of the progression rates consistent with those in adults, this being the largest age group in the population [5, 6].

Next, to explore each of the mechanisms hypothesised above, we modified the base model. Assumptions for each of these age-related mechanisms and their effects on the model structure are described in the following sections and summarised in Table 1.

**Table 1:** Summary of proposed age-related mechanisms

Mechanisms		Descriptions	Notation	Prior distribution	Parameters
					Definition (unit)
m0	Base model	No additional age-related mechanisms are incorporated.	*β*_*base*_	0.001–30	Infection rate (per year)
m1	Immune senescence	The risk of developing TB disease following recent or remote infection increases with age, potentially related to comorbidities and health-related behaviours.	*σ*^(*C*)^	0.1–1	Multiplier to baseline progression rates for children, compared to adults
			*σ*^(*E*)^	1–10	Multiplier to baseline progression rates for elders, compared to adults
m2	Declining transmission^a^	Frequent *Mtb* transmission in the past generated a substantial number of latently infected population, who become the elderly cases in the present day. A rapid decline of *Mtb* transmission happens recently because of improved living standard and health service.	*β*_*ini*_	0.001–30	Infection rate prior to *t*_0_ (per year)
			*β*_*end*_	0.001–30	Infection rate at 2017 (per year)
			*t*_0_	1957–2005	Beginning year of transmission decline
m3	Age-specific assortativity	Mixing between age groups lead to different infection tendencies, as a result of social activity and infectiousness. Elders are additionally restricted to mix with younger age groups in order to retain the high burden in the population.	*w*^(*A*)^	0.2–5	Connectivity weight of adults for mixing with others, compared to children
			*w*^(*E*)^	0.2–5	Connectivity weight of elders for mixing with others, compared to children
			*ε*	0.01–1	Isolation factor for mixing between elders and younger age groups

^a^We assumed uniform distributions for all prior parameters with the boundaries described. In models with the mechanism of age-specific assortativity (m3, m23, m13, and m123), *β*_*base*_, *β*_*ini*_, and *β*_*end*_ represent the infection rates within children, so those prior distributions were modified as 0.001–10

### Immune senescence (m1)

The first mechanism we proposed is that the breakdown of existing latent infection becomes progressively more likely in older age groups, as a result of a weakening immune system or age-related comorbidities (such as diabetes [18]). We therefore incorporated age-specific values for the per-capita primary progression rate ${\rho}_{f,m1}^{(a)}$ and reactivation rate ${\rho}_{s,m1}^{(a)}$. For simplicity, we assumed that both are related to the baseline progression rates in the base model (*ρ*_*f*, *m*0_, *ρ*_*s*, *m*0_) through age-specific multipliers *σ*^(*a*)^; that is,

$$ {\rho}_{f,m1}^{(a)}={\sigma}^{(a)}\times {\rho}_{f,m0} $$

$$ {\rho}_{s,m1}^{(a)}={\sigma}^{(a)}\times {\rho}_{s,m0}. $$

We further imposed the constraint *σ*^(*C*)^ ≤ *σ*^(*A*)^ ≤ *σ*^(*E*)^, relating to the multipliers for children, adults, and elders. As discussed above, because the baseline progression rates drawn from the literature represent adults, we have *σ*^(*A*)^= 1.

### Declining transmission (m2)

Next, we postulated that TB transmission was more intense in past generations before undergoing a rapid decline, as a result of improving living standards and health access over time. Under this hypothesis, most contemporary cases amongst the elderly therefore develop from reactivation of existing, latent infection. To capture the temporal trend in a simple way, we replaced the constant infection rate with a function of time *t*, structured as:

$$ {\beta}_{m2}(t)=\left\{\begin{array}{c}{\beta}_{ini},\kern16.5em if\ t\le {t}_0\\ {}{\beta}_{ini}+g\times \left(t-{t}_0\right),\kern9.5em if\ t>{t}_0\end{array}\right., $$

where *β*_*ini*_ represents the infection rate prior to the year *t*_0_, and afterwards, a gradient *g* determines how fast the rate linearly declines. The declining gradient *g* is modelled as (*β*_*end*_ − *β*_*ini*_)/(2017 − *t*_0_), where *β*_*end*_ represents the infection rate at 2017, and was restricted to be not less or equal than *β*_*ini*_. We assumed *t*_0_ to lie between 1957 and 2005, considering the availability of effective TB diagnosis and treatment services in Taiwan. With the incorporation of this ‘declining transmission’ mechanism, the force of TB infection was modified as:

$$ {\lambda}_{m2}(t)={\beta}_{m2}(t)\ \frac{PTB(t)}{N(t)}. $$

We note that an alternative mechanism is an increase in the rates at which TB cases are initiated on treatment, reflecting improvements in TB care. In Additional file 1 we present further analysis of this alternative mechanism, showing that it provides dynamical behaviour that is qualitatively the same as the ′*β*-mediated’ mechanism applied in the main analysis.

### Age-specific assortativity (m3)

The third mechanism we hypothesised is that a high TB burden is maintained in the elderly by preferential mixing amongst the elderly, and weaker mixing between the elderly and younger age groups to avoid the spillover of infection. We modelled this assortative mixing pattern by restructuring the force of TB infection for this mechanism as a function of time and age (*t* and *a*, respectively):

$$ {\lambda}_{m3}^{(a)}(t)={\beta}_{m3}{\sum}_j{\mathbf{M}}_{a,j}\ \frac{PTB^{(j)}(t)}{N^{(j)}(t)} $$

where the ‘mixing’ matrix **Μ**_*a*, *j*_ denotes the contact intensity between a susceptible person from age group *a* and an infectious person from age group *j*, and *PTB*^(*j*)^ and *N*^(*j*)^ are respectively the prevalence of pulmonary TB and the size of age group *j*. As a simple choice of **Μ**_*a*, *j*_, we assumed age groups mix in proportion to their respective weights *w*^(*a*)^, and further that the mixing between the elderly and the rest of the population is diminished by a factor *ε*. The mixing matrix therefore takes the following form:

$$ {\mathbf{M}}_{a,j}=\left[\begin{array}{ccc}{w}^{(C)}{w}^{(C)}& {w}^{(C)}{w}^{(A)}& \varepsilon {w}^{(C)}{w}^{(E)}\\ {}{w}^{(A)}{w}^{(C)}& {w}^{(A)}{w}^{(A)}& \varepsilon {w}^{(A)}{w}^{(E)}\\ {}\varepsilon {w}^{(E)}{w}^{(C)}& \varepsilon {w}^{(E)}{w}^{(A)}& {w}^{(E)}{w}^{(E)}\end{array}\right], $$

where we chose *w*^(*C*)^ = 1 for simplicity, thus interpreting *w*^(*A*)^ and *w*^(*E*)^ as the connectivity weights of adults and elders relative to children. *β*_*m*3_ can therefore be interpreted as the children-to-children infection rate.

### Model calibration

The age-related mechanisms were assessed individually, in pairwise combination and finally in full combination, to produce seven different mechanism models. All of these mechanism models and the base model were calibrated by comparing model outputs for annual TB treatment initiations against observed data for annual notifications. In particular, for reimbursement purposes, almost all TB cases are reported to the central surveillance system upon initiating TB treatment [19]. We used the aggregated notification rates over 1997–2004 [20] and age-specific notification rates over 2005–2016 [12] (the period when age-specific data was available). For simplicity, we assumed notification errors in diagnosis and reporting to be normally distributed. To reflect an improvement in TB data quality from 2005 onwards, we parameterised the normal distributions to allow for +/− 20% notification error over 1997–2004, and +/− 10% notification error over 2005–2016 (see Additional file 1: Table S.3.1 for resulting estimates of the standard deviations).

We adopted a Bayesian inference framework for model calibration, implemented by a Markov chain Monte Carlo (MCMC) method with the adaptive Metropolis algorithm [21, 22]. Uniform prior distributions for parameters on both the mechanisms and natural history of TB were used (Table 1 & Additional file 1: Table S2.2). We first calibrated each model with the Nelder-Mead simplex method using 100 initial parameter sets drawn from Latin Hypercube sampling; of these we assigned the two best-fit results as initial parameter values for independent MCMC chains and evaluated these chains for convergence. Each MCMC chain was run for 1,000,000 iterations and thinned to obtained 1000 posterior samples to reduce autocorrelation, after removing the burn-in iterations. We repeated the same process above for model calibration in each mechanism model.

### Model comparison

With the obtained posterior samples, we compared models on their ability to reproduce age disparities, as well as the gradually declining trend in TB burden observed in Taiwan. The indicators for age disparities include the average of adult-child and elder-child ratios of the notification rates over 2005–2016. For the temporal trend, the proportion of change over the same period in population-level TB notification rates was evaluated: (*rate*_2016_ − *rate*_2005_)/*rate*_2005_. Additionally, to quantify model fit, we calculated the deviance information criterion (DIC) as:

$$ DIC=2\overline{D}-D\left(\overline{\boldsymbol{\theta}}\right)=\overline{D}+\underset{pD}{\underbrace{\overline{D}-D\left(\overline{\boldsymbol{\theta}}\right)}} $$

where *D*(***θ***) is the deviance given posterior parameter set ***θ***. *D*(***θ***) is defined as −2 log *L*(***θ***), and *L*(***θ***) denotes the joint likelihood of the notification rates for calibration. As in the equation shown above, DIC can also be presented as a combination of measures for model fit ($\overline{\mathrm{D}}$) and complexity (*pD*). A smaller DIC value is favoured in model selection. In addition, we assessed the correlations between parameters and analysed the sources of incident TB cases, from recent infection, remote infection, and post-treatment recurrence.

### Sensitivity analyses

Above we mentioned that the ‘declining transmission’ mechanism was mediated by a rapid temporal decline in the infection rate, which may arise from improving living conditions. However, other approaches such as enhancing case detection and treatment access could also reduce opportunities for transmission. We used this alternative approach to model the ‘declining transmission’ hypothesis, by assigning a linear increasing trend to the treatment initiation rate (Additional file 1: Section S4). In addition, the models described above involve a simple representation of the ageing process. We assessed how a more sophisticated age structure would change the comparison of the proposed mechanisms. We constructed a cohort-ageing model, refining the age structure into single-year spans, and modelling the ageing process discretely, by shifting each cohort of age *a* to *a* + 1 (*a* = 0, 1, …, 99) at end of each simulated calendar year. Under this more realistic but computationally intensive age structure, we re-calibrated all mechanism models and the base model. To reduce the computation effort, we limited all the sensitivity analyses to the ‘best-fit’ parameter sets through the Nelder-Mead simplex method, without performing MCMC on these alternative models.

## Results

**Fig. 2:**
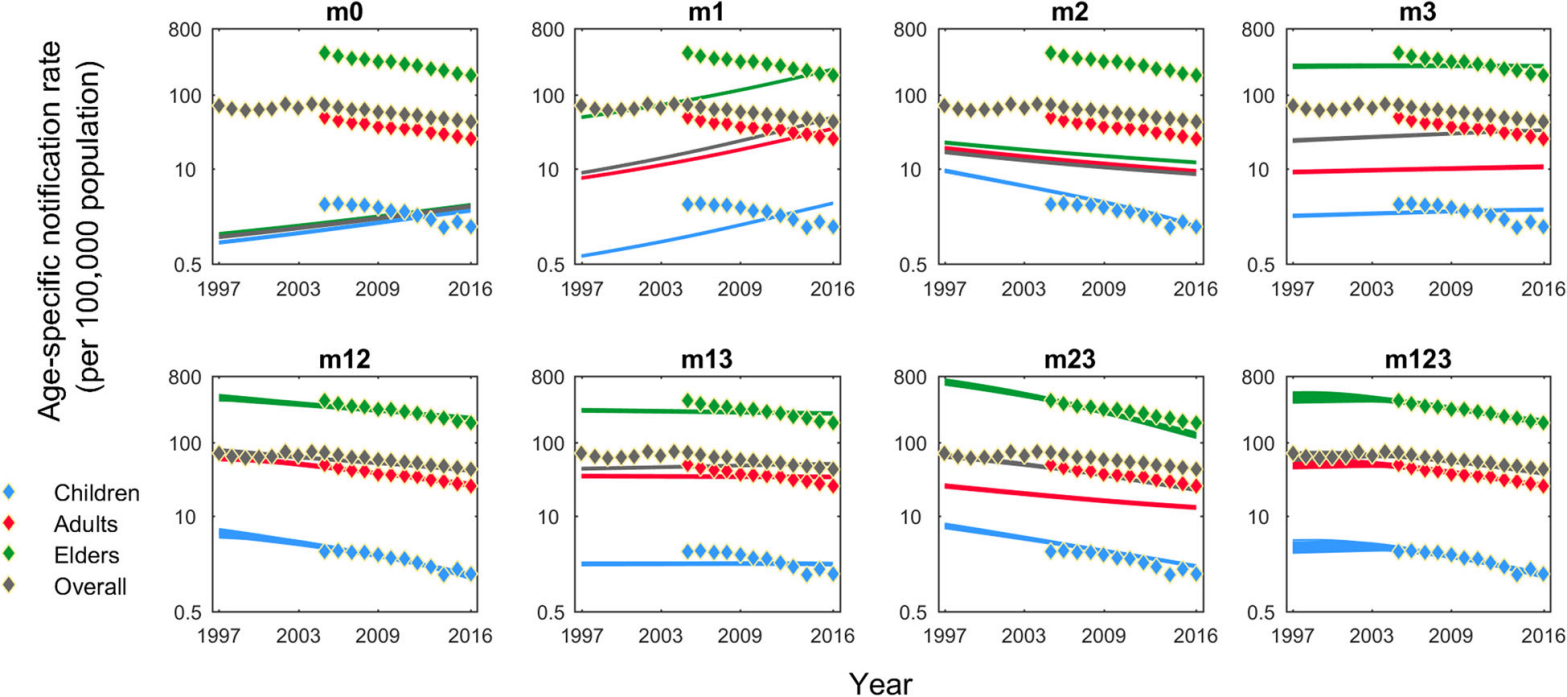
Calibration results of age-specific TB notification rates on a log scale. For each mechanism model, the TB notification rates over 1997–2016 are shown by children (blue), adults (red), elders (green), and aggregated population (dark grey). We present the model estimates from the 1000 posterior samples (lines), compared to the observed data (diamonds). Details of mechanism models are described in Table 1

**Fig. 3:**
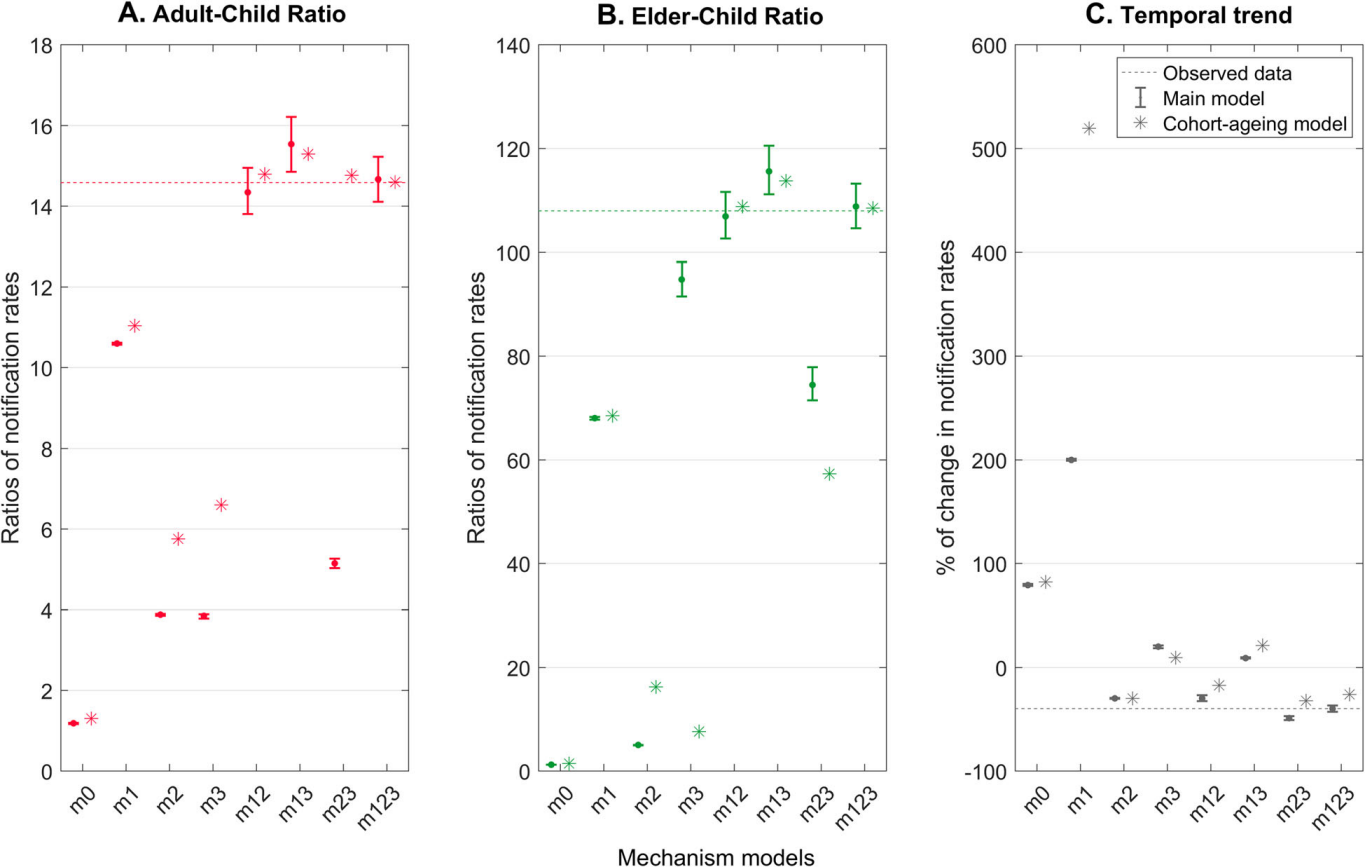
Age-specific ratios and temporal trend of TB notification rates. Age disparities of TB burden were evaluated by (**a**) adult-child and (**b**) elder-child ratios of the notification rates, and the temporal trend (**c**) was summarised by the proportion of change in the notification rates over 2005–2016. Dots and error bars show central points and 95% uncertainty intervals of the model estimates based on the 1000 posterior samples in the main analysis. Asterisk marks show the best-fit results obtained from the cohort-ageing models using the explicit ageing structure in sensitivity analysis. Horizontal dashed lines mark the observed data. Details of mechanism models are described in Table 1

Figure 2 shows a comparison of model estimates of the age-specific TB notification rates to the observed data on the log scale. Figure 3 shows quantitative indicators for how well the different models reproduce (i) age disparities, and (ii) recent, overall temporal trends in TB burden. Both figures illustrate that the base model (m0) was poorly calibrated, despite incorporating some age-specific characteristics of TB natural history, such as the proportions of pulmonary TB in total cases. By contrast, the ‘full’ model with all the three mechanisms (m123) best captured the age-specific notification rates over time, but we found that the two-mechanism model which incorporates the mechanisms of immune senescence and declining transmission (m12), also demonstrated a good fit to the adult-child and elder-child ratios of the notification rates. However, this model, without the age-specific assortativity mechanism, slightly underestimated the rate of decline in TB burden over 2005–2016.

**Fig. 4:**
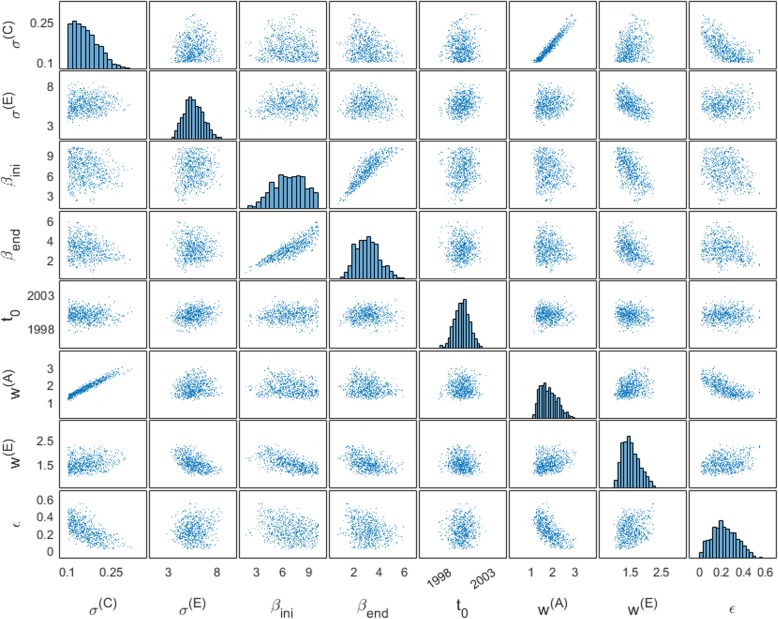
Scatter plot matrix of mechanism-related parameters in the ‘full model’. Scatter plots show distributions between each pair of posterior parameters, and histograms in the diagonal present the distribution of individual parameters. For clarity, only eight parameters specific to the three hypothesised mechanisms were included. Notations for the parameters are described in Table 1

None of the single-mechanism models could fully describe the age disparities in TB burden. Nonetheless, Fig. 2 provides some insight into the role that each of these mechanisms offers in matching the data. The mechanisms of immune senescence (m1) and of age-specific assortativity (m3) both act to capture the disparities of TB burden across age groups. Indeed, the model with the combined mechanisms of these two mechanisms (m13) led to an overestimate of the adult-child and elder-child ratios, reflecting their overlapping functions in capturing the age disparities (Fig. 4). On the other hand, the decreasing trend of TB burden could only be replicated by those models incorporating the declining transmission mechanism (m2). In models lacking this mechanism, an increasing TB burden results from an ageing population in this setting.

**Fig. 5:**
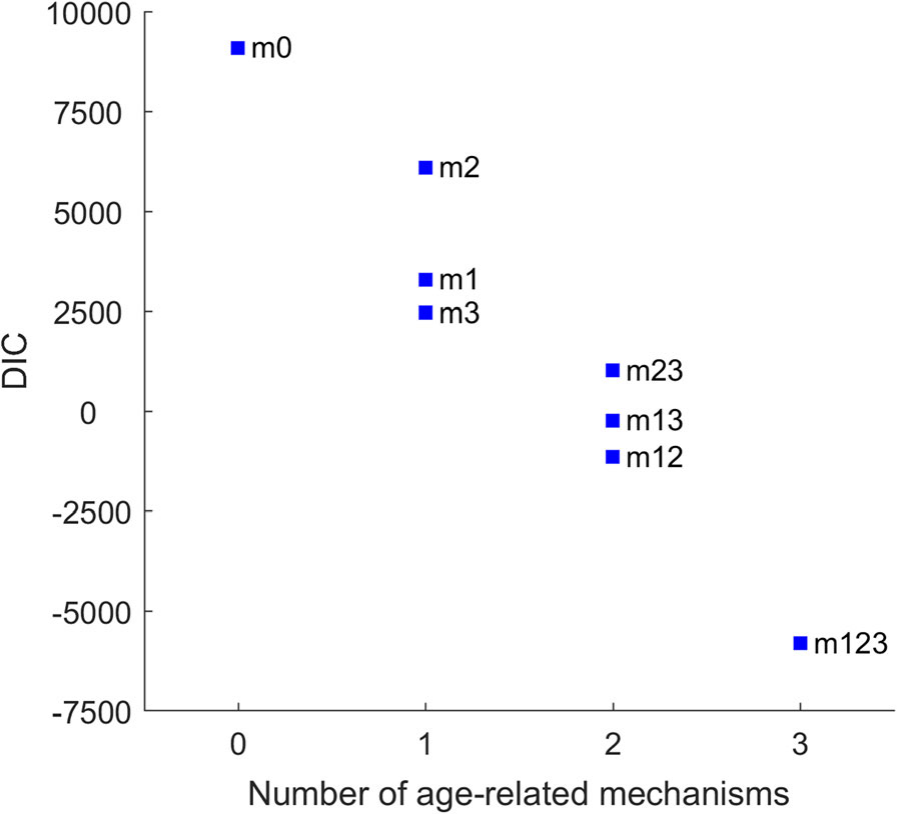
DIC Distribution by the number of age-related mechanisms included. The values of DIC decrease as more age-related mechanisms are included. Details of mechanism models are described in Table 1. Abbreviations: DIC-deviance information criterion

Table 2 presents values for DIC, log-likelihood, and the median and 95% credible intervals of key parameters for model calibration. Generally, DIC values dropped with increasing model complexity (Fig. 5), with the lowest (most favourable) DIC being observed in the ‘full’ model integrating all the three mechanisms. Separating the components of the DIC values, Table 3 reveals that the complexity measures had a limited effect on the overall performance while the fit measures are the dominating factors in model selection. Table 4 shows the attribution of incident TB cases to recent infection, remote infection, and post-treatment recurrence by age groups in the ‘full’ model (m123). Remote infection becomes a more important source of TB incidence with increased age and contributed to more than half of active cases in adults and elders. However, the role of recent infection is also important, accounting for an estimated of 47.3% (34.8–59.5%) among overall incidence.

**Table 2:** DICs, log-likelihood, and posterior parameters in each mechanism model

Model	Indicators		Posterior parameter distribution									
	DIC	logL	m0		m1		m2			m3		
			*γ*	*b*_*base*_	*σ*^(*C*)^	*σ*^(*E*)^	*b*_*ini*_	*b*_*end*_	*t*_0_	*w*^(*A*)^	*w*^(*E*)^	*ε*
m0	9090	− 4546 (− 4552,-4541)	1.78 (1.33, 2.41)	7.70 (6.09, 9.86)	-	-	-	-	-	-	-	-
**Single-mechanism model**												
m1	3288	− 1643 (− 1651, − 1638)	1.33 (1.32, 1.39)	5.35 (5.25, 5.55)	0.100 (0.100, 0.100)	9.99 (9.93, 10.0)	-	-	-	-	-	-
m2	3099	− 3048 (− 3055, − 3043)	2.96 (2.81, 3.03)	-	-	-	9.90 (9.47, 10.0)	2.02e-3 (4.05e-4, 8.67e-3)	1957.0 (1957.0, 1957.1)	-	-	-
m3	2455	− 1228 (− 1235, − 1223)	7.24 (5.87, 7.65)	15.2 (12.4, 16.4)	-	-	-	-	-	3.54 (3.46, 3.59)	5.00 (4.98, 5.00)	1.01e-2 (1.00e-2, 1.03e-2)
**Two-mechanism model**												
m12	− 1159	437 (432, 440)	1.98 (1.37, 2.54)	-	0.101 (0.100, 0.106)	9.06 (8.25, 9.82)	8.48 (6.29, 9.91)	0.593 (0.203, 1.06)	1986.0 (1977.3, 1989.9)	-	-	-
m13	− 242	118 (113, 122)	3.23 (1.36, 7.50)	10.9 (5.50, 23.7)	0.104 (0.100, 0.119)	1.02 (1.00, 1.09)	-	-	-	1.57 (1.46, 1.80)	4.80 (4.23, 4.99)	0.165 (0.127, 0.201)
m23	1012	− 504 (− 512, − 498)	7.39 (6.45, 7.66)	-	-	-	28.2 (24.8, 30.0)	4.64 (3.93, 5.23)	1957.2 (1957.0, 1957.8)	2.06 (1.98, 2.13)	5.00 (4.99, 5.00)	1.00e-2 (1.00e-2, 1.02e-2)
**Three-mechanism model (‘full’ model)**												
m123	− 5825	462 (458, 464)	5.94 (2.83, 7.58)	-	0.137 (0.103, 0.209)	5.50 (3.79, 7.62)	8.99 (4.34, 13.7)	4.02 (1.84, 6.55)	1999.9 (1998.3, 2001.5)	1.76 (1.25, 2.70)	1.49 (1.10, 2.10)	0.217 (0.035, 0.455)

The log-likelihood, and central estimates and 95% credible intervals (in brackets) of the key posterior parameters are presented for each mechanism model. For comparison across models, infection rates b_base_, b_ini_, and b_end_ reported in this table have been standardised by scaling the age-specific ‘mixing’ matrix so that the mean of its elements equals to 1. Details of parameters and mechanism models are described in the ‘Method’ section and Table 1. Abbreviations: DIC - deviance information criterion; logL – log-likelihood

**Table 3:** Fit and complexity measures of DIC by mechanism models

Model	DIC	Fit measure ($\overline{\mathrm{D}}$)	Complexity measure (*pD*)
m0	9090	9092	−2.31
m1	3288	3287	−0.78
m2	6099	6097	2.27
m3	2455	2457	−1.27
m12	−1159	− 873	− 285
m13	−242	− 236	−5.88
m23	1012	1009	3.66
m123	−5825	− 923	− 4902

Abbreviation: *DIC* deviance information criterion

Details of the mechanism models are described in Table 1

**Table 4:** Sources of incident TB cases in the ‘full’ model, 2016

Age groups	Recent infection	Remote infection	Post-treatment recurrence
Children	71.2% (60.0–79.9%)	27.0% (18.4–38.3%)	1.70% (1.51–1.89%)
Adults	48.2% (35.2–60.3%)	50.4% (38.3–63.4%)	1.44% (1.25–1.66%)
Elders	46.2% (33.0–60.8%)	52.6% (38.1–65.8%)	1.15% (0.98–1.32%)
Overall	47.3% (34.8–59.5%)	51.4% (39.2–63.9%)	1.30% (1.16–1.44%)

Proportions of TB cases developing from recent infection, remote infection, and post-treatment recurrence are reported by age groups. We calculated medians and intervals between 2.5th and 97.5th percentiles (in brackets) of the proportions from the 1000 posterior parameter sets of the ‘full’ model. Incident cases in the ‘recent infection’ category develop TB through primary progression within 2 years of infection, while those in the ‘remote infection’ category develop the disease through reactivation after two or more years of infection. TB cases occurring within 3 years of treatment completion, either from relapse or reinfection, are included in the ‘post-treatment recurrence’ category

Section S4 in Additional file 1 shows results of the sensitivity analysis on the ‘declining transmission’ mechanism (m2). When assuming that this mechanism was mediated by the treatment initiation rate rather than by the infection rate, we did not identify qualitative differences in the main calibration results. Similar to the effect of decreasing infection rate, improvement in treatment initiation tended to capture overall declining trends in TB burden, while contributing little to the age disparities of TB burden. In terms of the explicit ageing process, Fig. 3 demonstrates consistent findings resulting from the main models, on the relative roles of each mechanism in explaining the age disparities and temporal trend of TB burden, even though the calibrated parameters from the two ageing approaches show different distributions. This suggests that a model with simple age categories of children, adults, and elders is likely to be sufficient in capturing the age effect on TB dynamics in this setting.

## Discussion

Using an age-structured compartmental model, we explored the potential mechanisms contributing to the age disparities and recent temporal pattern observed in the TB notification rates in Taiwan. The age-specific TB burden was best described by the model with all the three proposed mechanisms together; amongst the more parsimonious models, the one capturing immune senescence and declining transmission (omitting age-specific assortativity) performed best in capturing the data. The sensitivity analysis suggested that the simple age structure incorporated in our model was appropriate in evaluating the effect of age-related mechanisms on TB burden.

Each age-related mechanism in this study performed different roles in capturing the TB burden in Taiwan. First, the ‘immune senescence’ mechanism appears to be most important in explaining age disparity, with its omission from the ‘full’ model resulting in the greatest increase in the DIC value (m23 vs m123 in Table 2). Parameter estimates in the full model suggest a 40-fold relative hazard of disease progression amongst the elderly, compared to children. Such disparities might be explained by the protective effect of BCG vaccination in children [23], combined - in older age groups - with the increased prevalence of risk factors such as diabetes [24] and smoking [25]. Overall, although our results suggest that immune senescence is indeed an important factor in the age disparities of TB burden, our quantitative estimates should be treated with caution: just as these estimates are sensitive to the inclusion of alternative mechanisms (comparing m1 with m123), they may be changed further by incorporating additional mechanisms that we have not covered here.

Second, while the ‘declining transmission’ mechanism was not able to capture the age disparities on its own (m2), it acted as a key influence in capturing the declining trend of TB burden. Indeed, models without this mechanism (m0, m1, m3 and m13) showed an overall increasing burden of TB in the recent decade, a trend driven by the ageing population in all the models (Fig. 3). In the full model m123, the initiation timing for this mechanism fell between 1995 and 2003. Besides the general improvement of living standards, this period coincides with the health sector reform that replaced a vertical TB control system with an integrated programme led by Taiwan Centers for Disease Control in 2002 [26]. In addition, the National Health Insurance was established in 1995 and its coverage exceeded 96% by 2000 [11]. Both of these major developments are likely to have reduced TB transmission intensity over time, and the subsequent scale-up of the Directly Observed Treatment, Short Course in 2006 and contact tracing in 2007 may have further contributed to the trend [27]. Resolving individual impacts of these various developments is outside the scope of the present study; nonetheless, our results present estimates that are consistent with this overall history of improvements in TB services in Taiwan.

Third, the mechanism of age-specific assortativity modified the force of infection and allowed heterogeneous risks of infection according to mixing between age groups. This appears to be the least influential mechanism for capturing recent age-specific trends, with its omission from the ‘full’ model resulting in the least increase in the DIC value (m12 vs m123 in Table 2). Nonetheless, an important question is how our hypothesis relates to existing sociological data for age-specific mixing. Diary-based contact surveys in Western Europe [28] suggest that the bulk of assortative mixing occurs between school-age children, and a subsequent study suggested a similar contact pattern in Taiwan [9]. These findings are consistent with a key aspect of our age-specific assortativity hypothesis, that the elderly have fewer reported contacts than other age groups. We also note that while such data have been helpful for acute infections such as influenza [9], it is unclear how applicable they may be to TB, where infection arises as a result of cumulative exposure over time. Hence, other forms of non-physical, sustained contacts (including shared air) may be more important in driving TB transmission [29].

As with any modelling studies, there are some limitations to note. Model performance may depend to some extent on the prior parameter ranges, for example with some of the single-mechanism models yielding parameter estimates at the extremes of these ranges (Table 2). However, we deliberately adopted wide prior ranges, to avoid unduly constraining the calibration: models requiring parameter values outside these ranges are therefore likely to be implausible. We assumed a simplified age structure, adopting just three age compartments with birth and death rates, and adjusting ageing rates to capture the demographic trends in Taiwan. Adopting fixed averaged per-capita hazards of ageing, as we have done here, can introduce unexpected population behaviour; nonetheless, we have compared the model against an alternative, considerably more complex model framework that incorporates explicit ageing. This structural sensitivity analysis suggests that our model results are at least qualitatively robust (Fig. 3). Moreover, we used the age-specific TB notification rates in Taiwan as the target data for calibration in this study. We believe that the data accurately reflect the age disparity in the TB burden in Taiwan, as a validation study cross-linking TB cases in the National TB Registry and National Health Insurance Database has confirmed the completeness and timeliness of the notification system [19]. Nonetheless, we note that TB cases could still be under-detected, especially for TB patients with atypical extrapulmonary symptoms. In future, other sources of data could help to further assess the mechanisms we have presented here. For example, Fig. S5.1 in Additional file 1 shows the age-specific LTBI prevalence that would be expected from single and combinations of the mechanisms. This illustrates the potential value of community-based TB infection surveys in distinguishing these mechanisms.

This modelling analysis yields potentially helpful insights into the important mechanisms behind the age disparities of TB burden in Taiwan. In particular, our study results suggest that control of TB in Taiwan should focus on prevention of disease progression (addressing the mechanism of immune senescence), in addition to control of transmission amongst the elderly (addressing the mechanism of age-specific assortative mixing) (Table 4). Likewise, in other intermediate TB burden settings showing similar age disparities [2, 3], an understanding of mechanisms behind the TB burden can be valuable in developing proper strategies that target specific age groups. In the meantime, further research on informing or validating parameters in different mechanisms will also be useful for projecting the age distribution of TB burden.

## Conclusions

Our findings show the age disparities and temporal trend of TB notification rates in Taiwan were best captured by incorporating all the three hypothesised mechanisms into the age-structured dynamic model. While the mechanism of *declining transmission* mainly explained the falling trend of TB burden in the recent decade, the other mechanisms of *immune senescence* and *age-specific assortativity* contributed to the wide disparity between children and elders. However, comparing different combinations of the mechanisms, we found that *immune senescence* played a more important role than *age-specific assortativity*, in driving the age disparities of TB burden*.* This revealed key implications for TB control in Taiwan and other similar contexts: strategies aiming for preventing disease progression can be impactful when combined with efforts to control ongoing transmission in the elderly. Further research on the detailed features of these age-related mechanisms will enhance the development of effective interventions.

## Supplementary information

**Additional file 1: Supplementary materials.** This document includes the model equations, parameter tables, calibration results, sensitivity analyses and relevant details of this study.

## Data Availability

The data generated or analysed during this study are included in this published article and its supplementary information file.

## Abbreviations

DIC: Deviance information criterion
MCMC: Markov chain monte carlo
TB: Tuberculosis
