# Supplementary material for: Explaining age disparities in tuberculosis burden in Taiwan: a modelling study
Source: BMC Infect Dis. 2020 Mar 4;20:191. doi: 10.1186/s12879-020-4914-2 (PMC7057673; doi:10.1186/s12879-020-4914-2)
Supplement: Supplementary file 1 — Additional file 1: Supplementary materials. This document includes the model equations, parameter tables, calibration results, sensitivity analyses and relevant details of this study. [file 12879_2020_4914_MOESM1_ESM.pdf]

Explaining age disparities in tuberculosis burden in Taiwan: a modelling study

Additional file 1: supplementary materials

S1. Age disparities in tuberculosis burden across countries

The age disparities in tuberculosis (TB) notification rates are particularly wide in Taiwan and Korea (Fig. S1.1), where an intermediate TB burden and a rapid population ageing trend coexist. In low TB burden countries such as the United Kingdom (UK) and the United States of America (US), greater age disparities are more likely seen in immigrants. Note that 95.8% of Asian and 76.8% of Hispanic/Latino TB cases in the US are foreign-born (1).

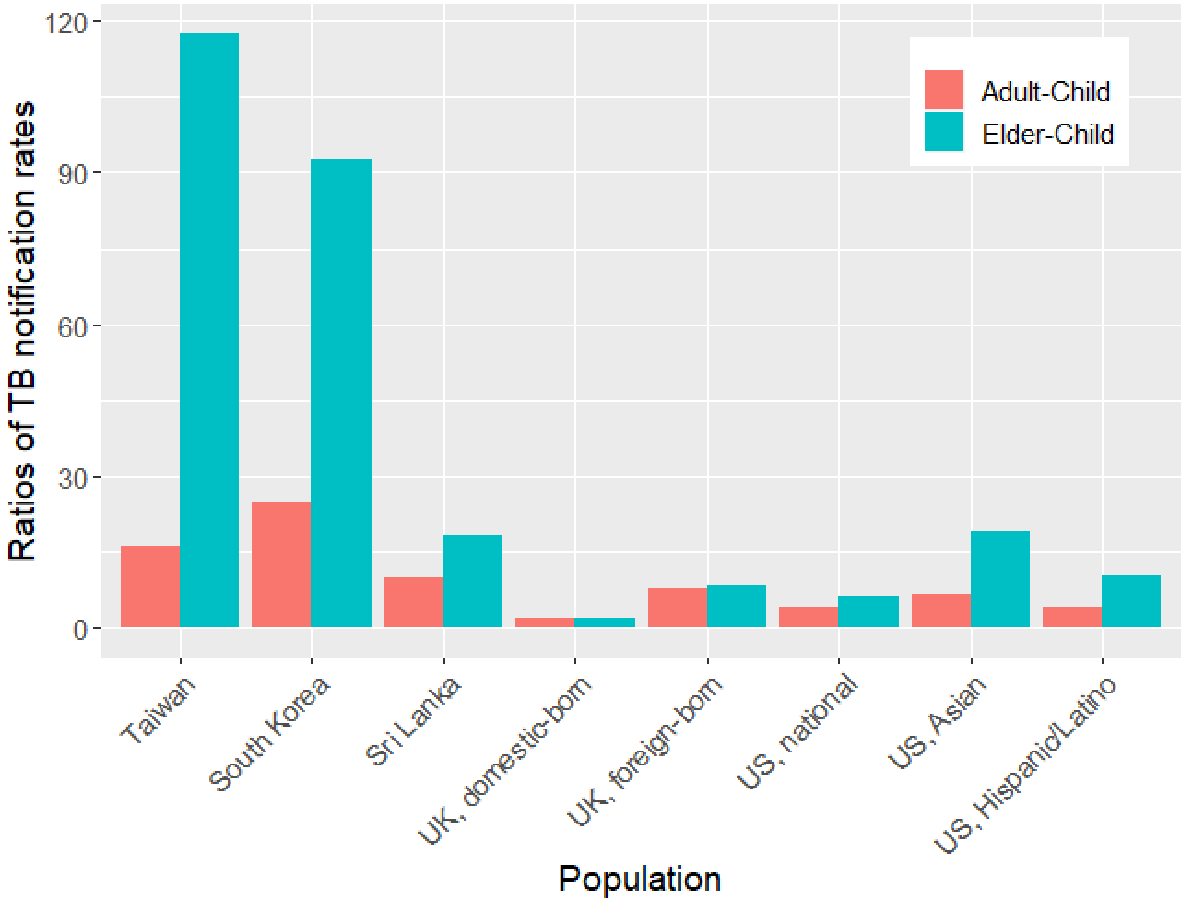

Fig. S1.1 Adult-Child and Elder-Child ratios of TB notification rates
The annual TB notification rates in several countries were extracted to calculate the relative ratios of adults (15-64 years old) and elders (≥ 65 years old), compared to children (< 15 years old). The reporting years were slightly different due to data availability (Taiwan (2), South Korea (3), UK (4) – 2016, Sri Lanka (5) – 2015, US (1) – 2017).

S2. Model description

The model included seven mutually-exclusive disease states, capturing the natural history of TB transmission (Table S2.1). The age structure was incorporated by further dividing each disease state into children (< 15 years old, $a = C$), adults (15-64, $a = A$), and elders (≥65 years, $a = E$). Model parameters for demographics and TB transmission are defined in Table S2.2.

Table S2.1 State symbols and descriptions.

Notation*	Description
$S^{(a)}$	Susceptible
$LF^{(a)}$	Latent-infected with fast progression
$LFRE^{(a)}$	Latent-reinfected with fast progression
$LS^{(a)}$	Latent-infected with slow progression
$PTB^{(a)}$	Active pulmonary TB
$ETB^{(a)}$	Active extrapulmonary TB
$TX^{(a)}$	Active TB cases under treatment
$R^{(a)}$	Recovered
$N^{(a)}$	Total population

*Superscripts (a) indicate age group a.

Table S2.2 Model parameters for demographics and TB transmission

Notation	Description	Unit	Value*	Sampling range**	Source
**Demographics**					
$\mu(t)$	Birth rate (time-dependent)	$year^{-1}$			(6)
$d_{allc}^{(a)}(t)$	All-cause death rate (time- and age-dependent)	$year^{-1}$			(6)
$\alpha_{in}^{(a)}$	Age group transition rates (flow-in)	$year^{-1}$	0<br>0.067<br>0.008	Fixed	Fitted
$\alpha_{out}^{(a)}$	Age group transition rates (flow-out)	$year^{-1}$	0.067<br>0.008<br>0	Fixed	Fitted
n	Initial population size	10^6	4.73	Fixed	Fitted
**TB transmission**					
$\lambda^{(a)}(t)$	Force of infection	$year^{-1}$		See Table 1.	
γ	Treatment initiation rate	$year^{-1}$		1.32– 7.67§§§	(7)
$\rho_{f,m0}$	Primary progression rate of adult population	$year^{-1}$	0.071	0.052 – 0.091§	(8)
$\rho_{s,m0}$	Reactivation rate of adult population	$year^{-1}$	0.00093	0.00072 – 0.00162§	(9)
θ	Protection from pervious infection		0.79	0.60 – 0.86§	(10)
$\upsilon_{LF \rightarrow LS}$	Stabilisation rate (the reciprocal of 2-year-duration in LF and $LREF$)	$year^{-1}$	0.50	Fixed	

$\upsilon_{R\rightarrow LS}$	Stabilisation rate (the reciprocal of 3-year-duration in R)	year^{-1}	0.33	Fixed	(11)
$\upsilon_{TX\rightarrow R}$	Treatment completion rate (the reciprocal of 6-month-duration of standard treatment)	year^{-1}	2	Fixed	
$\kappa_{etb}^{(a)}$	Proportion of extra-pulmonary cases among active TB cases		0.266<br>0.122<br>0.132	0.239 – 0.293[§§]<br>0.110 – 0.134[§§]<br>0.119 – 0.145[§§]	(12)
m_{ptb}	Mortality rate of pulmonary TB cases	year^{-1}	0.13	0.10 – 0.16[¶]	(13)
m_{etb}	Mortality rate of extra-pulmonary TB cases	year^{-1}	0.07	0.06 – 0.08[¶]	(13)
η	Natural cure rate of pulmonary and extra-pulmonary TB cases	year^{-1}	0.20	0.16 – 0.24[¶]	(13)
$\tau^{(a)}$	Proportion of treatment completion		1.000<br>0.929<br>0.664	0.900 – 1.000[§§]<br>0.836 – 1.000[§§]<br>0.598 – 0.730[§§]	(14)
$\varphi^{(a)}$	Recurrence rate	year^{-1}	0.0053<br>0.0046<br>0.0055	0.0048– 0.0058[§]<br>0.0041– 0.0051[§]<br>0.0050– 0.0061[§]	(11)

*Values of age-dependent parameters are listed in a top-down order of children (<14 years old), adults (15-64) and elders (≥65). Adjustments were made to match the age spans in our study.
**We assumed all the sampling ranges are uniformly distributed.
[§]Ranges were retrieved from the 95% intervals reported in the literature.
[§§]A ± 10% range of the central estimate was assumed because the programmatic data showed little variation over the recent decade. Note that the sum of treatment outcomes cannot be over 1.
[§§§]The sum of γ, m_{ptb}, and η, representing the reciprocal of the duration of infectiousness, was set to range from 1.5 to 7.3 months, based on a previous survey on healthcare seeking delay in 2005 (7) and an assumption on ≥ 80% of case detection rate.
[¶]Ranges were specified as ± 20% of central estimates.

To capture the demographic change over time, we applied an annual birth rate ($\mu(t)$) and age-specific all-cause death rates ($d_{allc}^{(a)}(t)$) to all compartments except for TB cases undergoing treatment, as their death rates were estimated separately by the programmatic data. The ageing process was simulated using per-capita rates of transition ($\alpha_{in}^{(a)}$, $\alpha_{out}^{(a)}$) between successive age groups, which is an approach relaxed in the sensitivity analysis (Fig. S6.1). In addition, the force of infection ($\lambda(t, a)$) and progression rates ($\rho_f^{(a)}$, $\rho_s^{(a)}$) are defined in the 'Methods' section of the main text, and their structures vary by the mechanisms involved.

$$\delta^{(a)} = \begin{cases} 1, & if\ a = C \\ 0, & if\ a \neq C \end{cases}$$

$$\mathrm{N} = \sum_a N^{(a)}$$

$$\frac{dS^{(a)}}{dt} = \alpha_{in}^{(a)} S^{(a-1)} + \delta^{(a)}\mu(t)N - \lambda^{(a)}(t)S^{(a)} - d_{allc}^{(a)}(t)S^{(a)} - \alpha_{out}^{(a)} S^{(a)}$$

$$\frac{dLF^{(a)}}{dt} = \alpha_{in}^{(a)} LF^{(a-1)} + \lambda^{(a)}(t)S^{(a)} - \upsilon_{LF\to LS} LF^{(a)} - \rho_f^{(a)} LF^{(a)} - d_{allc}^{(a)}(t)LF^{(a)} - \alpha_{out}^{(a)} LF^{(a)}$$

$$\frac{dLFRE^{(a)}}{dt} = \alpha_{in}^{(a)} LFRE^{(a-1)} + \lambda^{(a)}(t)LS^{(a)} - \upsilon_{LF\to LS} LFRE^{(a)} - (1-\theta)\rho_f^{(a)} LFRE^{(a)} - d_{allc}^{(a)}(t)LFRE^{(a)} - \alpha_{out}^{(a)} LFRE^{(a)}$$

$$\frac{dLS^{(a)}}{dt} = \alpha_{in}^{(a)} LS^{(a-1)} + \upsilon_{LF\to LS}\left(LF^{(a)} + LFRE^{(a)}\right) + \upsilon_{R\to LS} R^{(a)} - \lambda^{(a)}(t)LS^{(a)} - \rho_s^{(a)} LS^{(a)} - d_{allc}^{(a)}(t)LS^{(a)} - \alpha_{out}^{(a)} LS^{(a)}$$

$$\frac{dPTB^{(a)}}{dt} = \alpha_{in}^{(a)} PTB^{(a-1)} + \left(1-\kappa_{etb}^{(a)}\right)\rho_f^{(a)}\left[LF^{(a)} + (1-\theta)LFRE^{(a)}\right] + \left(1-\kappa_{etb}^{(a)}\right)\rho_s^{(a)} LS^{(a)} + \left(1-\kappa_{etb}^{(a)}\right)\varphi^{(a)} R^{(a)} - m_{ptb} PTB^{(a)} - \eta PTB^{(a)} - \gamma PTB^{(a)} - d_{allc}^{(a)}(t)PTB^{(a)} - \alpha_{out}^{(a)} PTB^{(a)}$$

$$\frac{dETB^{(a)}}{dt} = \alpha_{in}^{(a)} ETB^{(a-1)} + \kappa_{etb}^{(a)}\rho_f^{(a)}\left[LF^{(a)} + (1-\theta)LFRE^{(a)}\right] + \kappa_{etb}^{(a)}\rho_s^{(a)} LS^{(a)} + \kappa_{etb}^{(a)}\varphi^{(a)} R^{(a)} - m_{etb} ETB^{(a)} - \eta ETB^{(a)} - \gamma ETB^{(a)} - d_{allc}^{(a)}(t)ETB^{(a)} - \alpha_{out}^{(a)} ETB^{(a)}$$

We assumed the outcome for TB patients who initiate antibiotic treatment to be binary: completion or death (14). Given the observed proportions of treatment completion ($\tau^{(a)}$) and the fixed duration of standard treatment (transformed into the treatment completion rate $\upsilon_{TX\to R}$), we could derive the age-specific death rates during treatment ($d_{tx}^{(a)}$) according to the equation:

$$1 - \tau^{(a)} = \frac{d_{tx}^{(a)}}{d_{tx}^{(a)} + \upsilon_{TX\to R}}.$$

$$\frac{dTX^{(a)}}{dt} = \alpha_{in}^{(a)} TX^{(a-1)} + \gamma\left(PTB^{(a)} + ETB^{(a)}\right) - \upsilon_{TX\to R} TX^{(a)} - d_{tx}^{(a)} TX^{(a)} - \alpha_{out}^{(a)} TX^{(a)}$$

$$\frac{dR^{(a)}}{dt} = \alpha_{in}^{(a)} R^{(a-1)} + \eta\left(PTB^{(a)} + ETB^{(a)}\right) + \upsilon_{TX\to R} TX^{(a)} - \varphi^{(a)} R^{(a)} - \upsilon_{R\to LS} R^{(a)} - d_{allc}^{(a)}(t)R^{(a)} - \alpha_{out}^{(a)} R^{(a)}$$

S3. Model calibration

We calibrated each of the mechanism models to capture the aggregated notification rates over 1997-2004 and age-specific rates over 2005-2016. These calibration targets were assumed to be normally distributed, with the estimated standard deviations to reflect 20% or 10% of the notification errors, according to the data quality (Table S3.1).

Table S3.1 Means and standard deviations for the normally distributed calibration targets*

**Aggregated notification rates** (+/- 20% of notification errors)								
Year	1997	1998	1999	2000	2001	2002	2003	2004
Mean	70.7	64.6	61.1	62.4	64.7	74.4	66.5	74.0
Standard deviation	7.2	6.6	6.2	6.4	6.6	7.6	6.8	7.5
**Age-specific notification rates** (+/- 10% of notification errors)**								
Year	2005	2006	2007	2008	2009	2010	2011	2012
Mean	3.2<br>49.5<br>373.0	3.3<br>45.6<br>338.5	3.2<br>42.1<br>314.9	3.1<br>40.5<br>306.4	2.9<br>36.9<br>284.7	2.7<br>36.3<br>279.3	2.6<br>34.9<br>258.8	2.3<br>33.9<br>246.1
Standard deviation	0.2<br>2.5<br>19.0	0.2<br>2.3<br>17.3	0.2<br>2.1<br>16.1	0.2<br>2.1<br>15.6	0.1<br>1.9<br>14.5	0.1<br>1.9<br>14.2	0.1<br>1.8<br>13.2	0.1<br>1.7<br>12.6
Year	2013	2014	2015	2016				
Mean	2.0<br>30.8<br>227.2	1.6<br>29.6<br>212.0	1.9<br>27.2<br>195.7	1.6<br>25.7<br>185.0				
Standard deviation	0.1<br>1.6<br>11.6	0.1<br>1.5<br>10.8	0.1<br>1.4<br>10.0	0.1<br>1.3<br>9.4				

*All parameters for the normally distributed notification rates are presented with the unit of per 100,000 population.

**Parameters for age-specific notification rates are shown in the order of children, adults, and elders from top to bottom.

As described in the main text, we adopted a Bayesian inference framework in model calibration, with adaptive Markov chain Monte Carlo (MCMC) method. For each mechanism model, we ran two chains of 300,000 iterations with different starting points. Traces of the two MCMC chains were juxtaposed for evaluation of convergence (Fig. S3.1). We then extracted 50,000 iterations from the well-mixed traces and extended them into a long MCMC chain with additional 1,000,000 iterations. After thinning the long chain, we obtained 1000 posterior parameter sets (Fig. S3.2) for the following comparison on the performance of mechanism models.

In the full model incorporated with all the three hypothesised mechanisms, we examined the correlations of the posterior parameters (Fig. S3.3). Among the mechanism-related parameters, a positive correlation was observed between the initial (β_{ini}) and current infection rates (β_{end}), reflecting that the model tended to hold the slope of transmission decline within a certain range. In addition, correlations between parameters of different mechanisms existed. We found a positive relationship between the progression multiplier of children ($\sigma^{(C)}$) and the mixing weight of adults ($w^{(A)}$). This relationship was likely to maintain the age disparities of TB burden between children and adults. On the other hand, among the elderly-specific parameters, there was an inverse relationship between the progression multiplier ($\sigma^{(E)}$) and the mixing weight ($w^{(E)}$). Although the inverse relationship seemed less explicit, it implied the competing roles of the immune senescence (m1) and age-specific assortativity (m3) mechanisms in capturing the age disparities of TB burden.

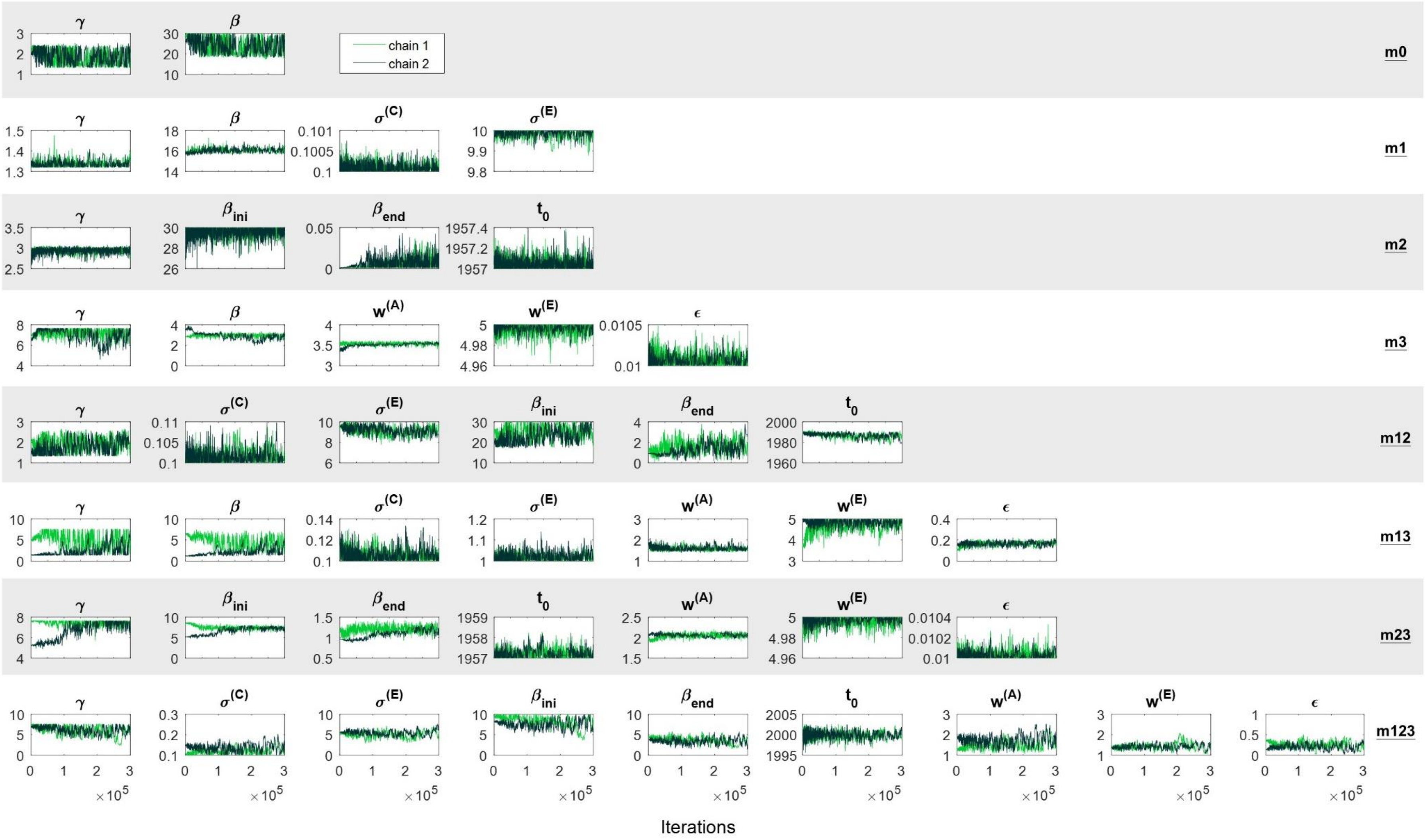

Fig. S3.1 MCMC traces for mechanism-related parameters

In each row, traces for mechanism-related parameters in each model are shown. Light and dark green lines are used to denote two different MCMC chains. Notations of the mechanisms and parameters are described in Table 1 and Table S2.2.

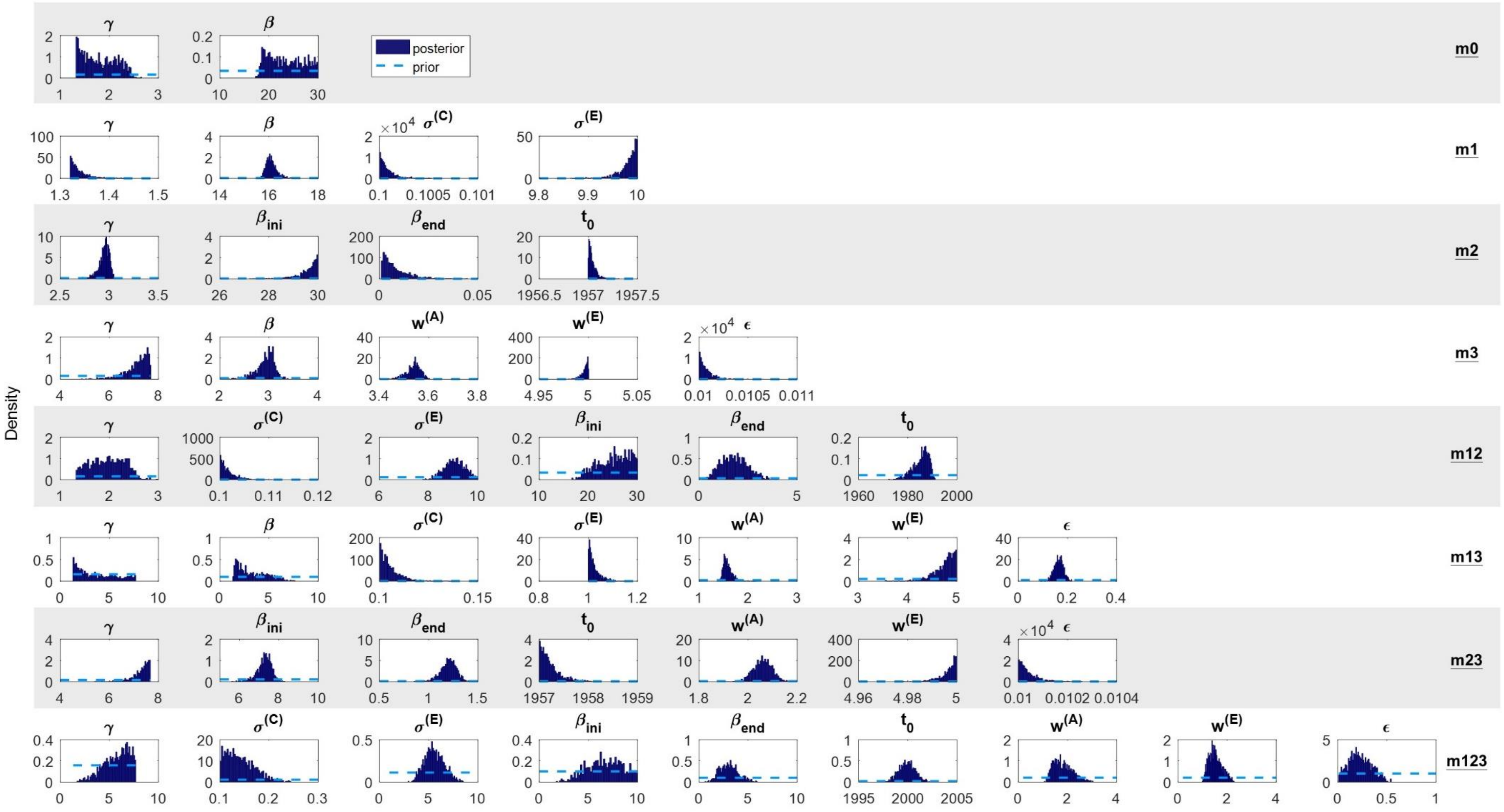

Fig. S3.2 Density of prior and posterior distributions for mechanism-related parameters
By row, we show prior (horizontal dashed lines) and posterior (dark bars) density distributions of parameters based on the 1000 thinned samples in each mechanism model. For clarity, only parameters specific to each mechanism are included. Notations of the mechanisms and parameters are described in Table 1 and Table S2.2

S4. Alternative approach for the 'declining transmission' hypothesis

As a sensitivity analysis, we captured the declining transmission mechanism by varying the treatment initiation rate (γ), instead of the infection rate (β). This alternative approach represents the improvement of TB services in case detection and treatment, reducing opportunities for transmission by shortening the duration of infectiousness. To assess this alternative assumption, we modified the treatment initiation rate as a function of time t, structured as:

$$\gamma'_{m2}(t) = \begin{cases} \gamma'_{ini}, & if\ t \leq t'_0 \\ \gamma'_{ini} + g' \times (t - t'_0), & if\ t > t'_0 \end{cases}$$

where γ'_{ini} represents the constant rate before time t'_0, and afterwards, the rate changes linearly with a slope g'. In order to create a model structure equivalent to that in the main analysis, we defined g' as followed:

$$g' = \frac{\gamma'_{end} - \gamma'_{ini}}{2017 - t'_0}$$

where γ'_{end} is the treatment initiation rate at 2017. We assumed a monotonically increasing trend of treatment initiation rates, thus applying the constraints $\gamma'_{end} \geq \gamma'_{ini}$ and $g' \geq 0$. As in the main model, we assumed t'_0 to occur between 1957 and 2005. The transmission parameter β'_{m2} under this alternative approach was set to be a fixed value over time.

We concentrated on the outputs of the single-mechanism model of declining transmission (m2). The same calibration target was used, but the model was optimised with the Nelder-Mead simplex method based on ten Latin Hypercube samples. Among the ten samples, the calibration result with the maximised likelihood was used to compare with the main assumption for model performance in capturing the age disparities and temporal trend of TB burden (Fig. S4.1).

The alternative approach using the time-varying treatment initiation rate did not change the conclusion that how the mechanism of declining transmission contributed to the age disparities and temporal trend of TB notification rates (Fig. S4.1). The age disparities between children and elders could not be replicated by either assumption of the declining transmission mechanism (Fig. S4.2). However, the assumption of a decreasing infection rate in the main model showed better model performance in capturing the temporal trend of TB burden, compared to the alternative assumption.

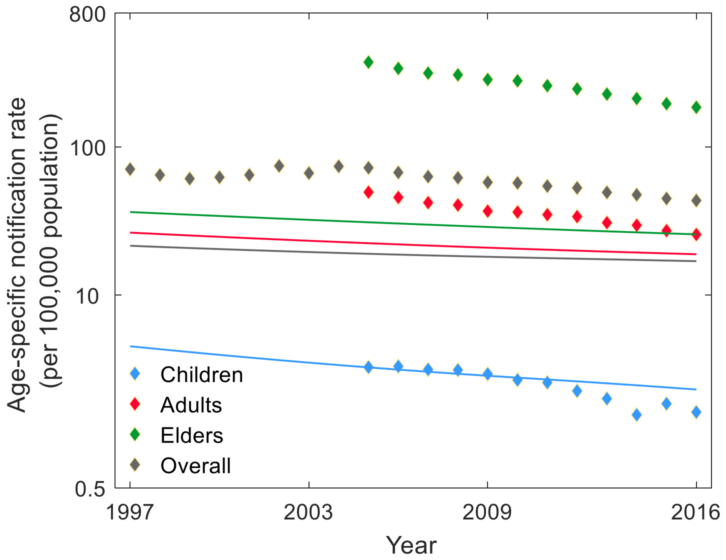

Fig. S4.1 TB notification rates calibrated by increasing treatment initiation rate
Solid lines demonstrated the calibration results of the TB notification rates in children (blue), adults (red), elders (green), and aggregated population (grey) over 1997-2016. Solid diamonds marked the data points for model calibration.

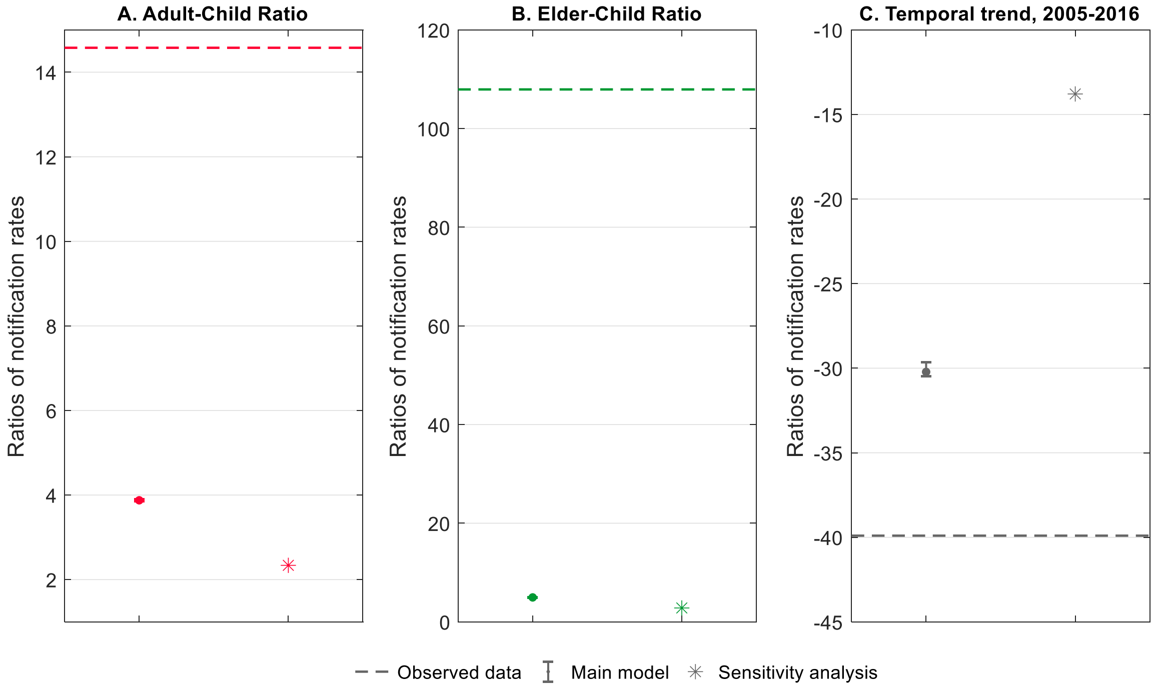

Fig. S4.2 Age disparities and temporal trend of the TB burden under different assumptions of the declining transmission mechanism (m2)
Asterisks show the results of model performance under the assumption of an increasing treatment initiation rate, whereas dots and error bars demonstrate the central estimates and uncertainty intervals under the assumption of a decreasing infection rate in the main model. The horizontal dashed lines represent calibration targets obtained from the observed data.

S5. Estimated age-specific prevalence of latent TB infection (LTBI)

A contact survey in Taiwan (15) reported 23% and 38% of positive results of TB infection by interferon-γ release assay and tuberculin skin test, respectively, among the elderly. While the survey data may partly inform the possible range of age-specific LTBI prevalence, further community-based data would be increasingly helpful in understanding of between-variation of the proposed mechanism models.

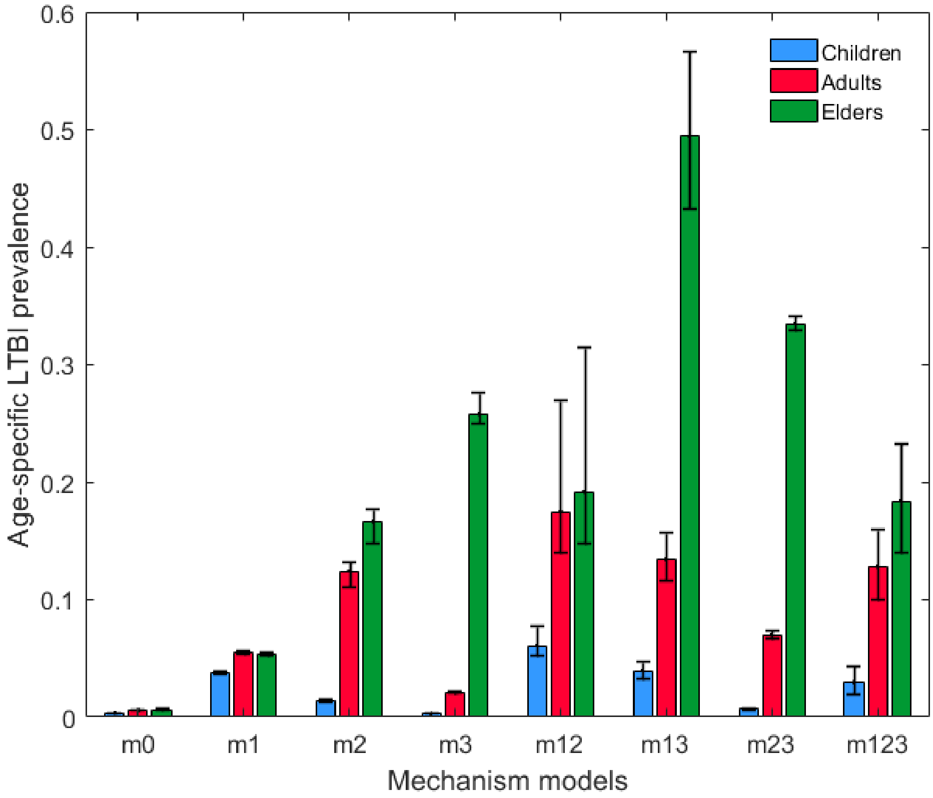

Fig. S5.1 Age-specific LTBI prevalence in 2016
By each mechanism model, the bars show the central estimates of LTBI prevalence in children (blue), adults (red), and elders (green). The corresponding 95% uncertainty intervals were added to the top of each bar.

References

1. Centers for Disease Control and Prevention. Reported Tuberculosis in the United States, 2017. Atlanta, United States; 2018.
2. Taiwan National Infectious Disease Statistics System: tuberculosis [Internet]. 2019 [cited 13 February 2019]. Available from: http://nidss.cdc.gov.tw/ch/SingleDisease.aspx?dc=1&dt=3&disease=010.
3. Go U, Park M, Kim UN, Lee S, Han S, Yang J, et al. Tuberculosis prevention and care in Korea: Evolution of policy and practice. J Clin Tuberc Other Mycobact Dis. 2018;11:8.
4. Public Helath England. Tuberculosis in England: 2017. London; 2017.
5. Medical Statistics Unit (Sri Lanka). Annual Health Bulletin, 2015. Colombo, Sri Lanka: Ministry of Health, Nutrition and Indigenous Medicine; 2017. Report No.: ISBN: 978-955-702-045-7.
6. Taiwan Department of Household Registration. Population Statistics.
7. Chiang CY, Chang CT, Chang RE, Li CT, Huang RM. Patient and health system delays in the diagnosis and treatment of tuberculosis in Southern Taiwan. Int J Tuberc Lung Dis. 2005;9(9):1006-12.
8. Vynnycky E, Fine PE. The natural history of tuberculosis: the implications of age-dependent risks of disease and the role of reinfection. Epidemiol Infect. 1997;119(2):183-201.
9. Shea KM, Kammerer JS, Winston CA, Navin TR, Horsburgh CR, Jr. Estimated rate of reactivation of latent tuberculosis infection in the United States, overall and by population subgroup. Am J Epidemiol. 2014;179(2):216-25.
10. Andrews JR, Noubary F, Walensky RP, Cerda R, Losina E, Horsburgh CR. Risk of progression to active tuberculosis following reinfection with Mycobacterium tuberculosis. Clin Infect Dis. 2012;54(6):784-91.
11. Lee PH. Diabetes mellitus increases the risk of recurrent tuberculosis: a population-based nested case-control study: National Taiwan University; 2012.
12. Taiwan Centers for Disease Control. Tuberculosis Annual Report 2000. 2002.
13. Tiemersma EW, van der Werf MJ, Borgdorff MW, Williams BG, Nagelkerke NJ. Natural history of tuberculosis: duration and fatality of untreated pulmonary tuberculosis in HIV negative patients: a systematic review. PloS one. 2011;6(4):e17601.
14. Taiwan Centers for Disease Control. Taiwan Tuberculosis Control Report 2016. 2017. Contract No.: ISSN 1997-1109.
15. Taiwan Centers for Disease Control. Policy communication: preventive thepary for latent TB infection in Taiwan 2016 [Available from: https://www.cdc.gov.tw/professional/info.aspx?treeid=89b930c89c1c71cf&nowtreeid=f1d017ce6dffbf55&tid=1DCECA3C62171B35.
